# Multimodal treatment and immune checkpoint inhibition in sinonasal mucosal melanoma: real-world data of a retrospective, single-center study

**DOI:** 10.1007/s00405-023-08015-8

**Published:** 2023-06-05

**Authors:** Agmal Scherzad, Manuel Stöth, Till J. Meyer, Lukas Haug, Thomas Gehrke, Bastian Schilling, Svenja Meierjohann, Matthias Scheich, Rudolf Hagen, Anja Gesierich, Stephan Hackenberg

**Affiliations:** 1 Department of Otorhinolaryngology, Plastic, Aesthetic and Reconstructive Head and Neck Surgery, University Hospital Würzburg, Josef-Schneider-Str. 11, 97080 Würzburg, Germany; 2 Department of Dermatology, Venerology and Allergology, University Hospital Würzburg, 97080 Würzburg, Germany; 3 Institute of Pathology, University of Würzburg, 97080 Würzburg, Germany; 4 Comprehensive Cancer Center Mainfranken, University Hospital Würzburg, Würzburg, Germany; 5 Department of Otorhinolaryngology-Head and Neck Surgery, RWTH Aachen University Hospital, 52074 Aachen, Germany

**Keywords:** Melanoma, Sinonasal, Immune checkpoint inhibition, Immunotherapy, Surgery, Mutation status

## Abstract

**Purpose:**

Local failure and distant metastases occur frequently in sinonasal mucosal melanoma (SNMM). Response rates to chemotherapy are low and targetable mutations are rarely detected. However, there is increasing data indicating efficacy of immune checkpoint inhibition (ICI). The aim of this retrospective monocenter study was to assess the mutational landscape and to evaluate the outcome of surgical treatment and ICI in SNMM in a real-world setting.

**Methods:**

Thirty-eight SNMM patients being treated between 1999 and 2020 at our institution were retrospectively reviewed. Survival curves were generated according to Kaplan–Meier and compared by the log-rank test.

**Results:**

Local failure was seen in 60% of patients treated in a curative intent. Overall, 24% of all patients suffered from regional and 66% from distant metastases. Next generation sequencing revealed mutations of BRAF, NRAS and KRAS. One out of three patients treated with a primary ICI showed a complete response (CR) and two showed progressive disease. Eleven patients received ICI as a palliative treatment. CR could be observed in three patients and stable disease in one patient. In the whole study population, the 5-year overall survival rate (OS) was 26%. OS was better for patients who received ICI during the course of disease.

**Conclusions:**

Recurrences and distant metastases are frequent in SNMM. Durable CR could be observed after primary and palliative ICI. Therefore, ICI in a palliative, adjuvant or even neoadjuvant setting might play a promising role in SNMM therapy while targetable mutations are rarely detected.

## Introduction

Sinonasal mucosal melanoma (SNMM) is a rare and aggressive disease representing only 4% of all sinonasal malignancies and 1.4% of all melanomas [1]. SNMM are summarized with the melanomas of the oral cavity as melanomas of the upper aerodigestive tract and are subsumed in the literature with melanomas of the genitoanal area as mucosal melanomas. Mucosal melanomas can occur in all mucosal epithelia, with the female genital tract being the most common site affected, followed by the anorectal area, the nasal cavity and paranasal sinuses [2]. Interestingly, mucosal melanoma differs significantly from other melanoma subtypes in terms of epidemiology, etiology and molecular characteristics [3]. Most common mutated genes for mucosal melanoma are found in NRAS, BRAF, NF1, KIT, SF3B1, TP53, SPRED1, ATRX, HLA-A and CHD8 [4]. However, alterations in NRAS, BRAF, TP53 and CDKN2A are more frequently found in cutaneous malignant melanoma (CMM) [5]. In contrast to its cutaneous counterpart, sun exposure is not associated with SNMM [6]. A familial predisposition is not described. Potential risk factors for the development of these tumors are unknown [7]. The incidence of mucosal melanomas has its peak in the age range from 60 to 80 years [8]. Prognosis of SNMM is poor with a 5-year overall survival (OS) between 26% and 45% [9]. The risk of local recurrence or progression is increased for R1/2 resection, advanced primary tumor extent or the presence of locoregional disease [10, 11]. The benefit of clear margins on overall survival is not clear and, therefore, still considered controversial [10–14].

SNMM are often diagnosed with delay due to non-specific symptoms such as epistaxis. For this reason, patients suffering from SNMM often present with locally advanced disease compared to CMM, which in turn contributes to a poor prognosis in SNMM [15]. At time of diagnosis, around 20% of patients present with regional lymph node disease and up to 10% with distant metastases. Liver, lungs and distant lymph nodes are the organs most frequently affected [9]. Most of these tumors occur in the nasal cavity and fewer arise in the paranasal sinuses [13]. The delicate anatomical localization of SNMM and its close relationship to adjacent organs such as brain, orbit, and the vasculature accounts for a possible invasion into these structures. Therefore, an extensive resection with sufficient safety margins is challenging, sometimes impossible, and might account for a significant treatment associated morbidity. This is why gross tumor resection (GTR), defined as the removal of all tumor parts without visual residual enhancing tumor, is the standard treatment for most sinonasal solid malignancies. The multimodal concept including surgery enhanced by an adjuvant radiotherapy is a widely accepted standard of care for the treatment of SNMM [16]. Though, the benefit of adjuvant radiotherapy on local control and survival in mucosal melanoma is still under debate [17]. New therapeutics such as immune checkpoint inhibitors (ICI) implemented in the treatment of mucosal melanoma are promising. ICI such as Programmed cell death protein 1 (PD-1) and cytotoxic T-lymphocyte-associated protein 4 (CTLA-4) inhibitors are well-established substances in the treatment of CMM [18] and show activity in mucosal melanoma as well. However, data on ICI therapy in mucosal melanoma are significantly less than in CMM. One reason is that the subgroup of SNMM is excluded in the majority of recent prospective clinical trials, causing a need for further data. This need could be met by ongoing studies, testing ICI in various combinations and settings. As yet, the available evidence for ICI is based on single case reports, retrospective analyses and small study subpopulations in clinical trials. In two published cases of metastatic mucosal melanoma, a complete remission was seen following Nivolumab, Ipilimumab plus Denosumab combination therapy [19] and Nivolumab monotherapy [20], respectively. In a pooled analysis from six prospective clinical trials, the data of 157 patients with unresectable or metastatic mucosal melanoma were analyzed. Objective response rates (ORR) of 8.3% (95% CI 1.8–22.5%) for ipilimumab, 23.3% (95% CI 14.8–33.6%) for nivolumab and 37.1% (95% CI 21.5–55.1%) for the combination therapy with nivolumab and ipilimumab were reported. Survival data were not presented [21]. A subgroup analysis of 63 patients from the prospective, single-arm Phase II CheckMate 172 study included 63 patients with advanced mucosal melanoma treated with nivolumab who had previously failed ipilimumab therapy. These patients showed a median OS of 11.5 months (95% CI 6.4–15.0%) with an 18-month OS rate of 31.5% [22]. In a post hoc analysis of different prospective studies (KEYNOTE-001, -002 and -006) investigating the efficacy and/or safety of pembrolizumab in advanced mucosal melanoma, a median OS of 11.3 months (95% CI 7.7–16.6%) was reported. The ORR was 22% in ipilimumab-naive patients and 15% in ipilimumab-pretreated patients with a median duration of response of 27.6 months in the total cohort [23]. In a multi-institutional retrospective cohort analysis of 35 patients with mucosal melanoma receiving anti-PD-1 therapy (nivolumab or pembrolizumab) after pretreatment with ipilimumab, an ORR of 23% (95% CI 10–40%) and a median progression-free survival (PFS) of 3.9 months after a median follow up of 10.6 months were reported [24].

Thus, patients with advanced mucosal melanoma may respond to PD-1 based ICI with regard to OS, but the ORR is significantly lower than in CMM. The monotherapy with ipilimumab shows a very low activity with an ORR of less than 10% [21]. In all regimes, the safety and rate of adverse events was similar to CMM. In comparison to chemotherapy, anti-PD-1 therapy also seems to have a more favorable benefit-risk ratio [25].

In this study, we retrospectively obtained epidemiological, clinicopathological, molecular and survival data of 38 SNMM patients treated at our institution. As there is an urgent need for more clinical data, a special focus of the analysis was set on ICI treatment given in a primary, palliative and adjuvant setting.

## Materials and methods

Thirty-eight SNMM patients being treated between 1999 and 2020 at our institution were retrospectively reviewed. Data was obtained from electronic medical records. Recurrence free survival (RFS) was calculated as the time from primary treatment to recurrence, OS was calculated from the date of diagnosis. Residual disease was defined as a persistent malignancy at any site detected within 3 months after completing first line treatment in a curative intent. Any malignancy diagnosed after 3 months was defined as recurrent disease. TNM categorization was performed retrospectively according to the eighth Edition of the UICC TNM Classification of Malignant Tumors.

Mutational analyses were carried out using Next Generation Sequencing (Ion Torrent PGM, Thermo Fisher, Waltham, Massachusetts, USA) at the local Institute of Pathology since March 2017 and Sanger Sequencing was used before. The Ion AmpliSeq Cancer Hotspot Panel v2 (Thermo Fisher) was used. The number and types of genes sequenced differed between patients. This is mostly due to the long period of 21 years evaluated.

All data were analyzed using Prism 5.01 (GraphPad Software, GraphPad Software Inc., San Diego, California, USA). Survival curves were generated according to Kaplan-Meier. Statistical significance was calculated by the log-rank test. A *p-*value < 0.05 was considered to be significant.

## Results

Within the study population of SNMM patients, the median age at time of diagnosis was 71 years with a predominance in female patients (58%). Most common symptoms upon first presentation were epistaxis (66%) followed by nasal obstruction (34%), rhinorrhea (8%) and facial pain (5%). Only nine patients (24%) had a history of tobacco consumption.

The primary site of SNMM was the nasal cavity (84%) with or without infiltration of the paranasal sinuses. There were six cases of SNMM solely affecting the paranasal sinuses: five cases in the maxillary sinus and ethmoid and one tumor arising from the frontal sinus. Tumor stage, localization, extension and histopathological information are shown in Table 1. At time of diagnosis, around 25% of the tumors were locally very advanced with 10 cases suffering from skull base affection, including six patients with dural and one with cerebral infiltration. Furthermore, 10 patients presented with orbital tumor invasion. Locoregional lymph node disease was present in five patients (13%) and distant metastases in four patients (11%) at time of diagnosis.

**Table 1:** Tumor staging, localization, histopathological and mutational data

Parameter	Number (%)	Parameter	Number (%)
Staging		Infiltration	
T3	19 (50)	Skull base	10 (26)
T4a	9 (24)	Dura	6 (16)
T4b	10 (26)	Intracerebral	1 (3)
N0	33 (87)	Orbit	10 (26)
N1	5 (13)	Mutation analysis	
M0	30 (78)	BRAF (nonV600)	1 of 22
M1	4 (11)	c-KIT	0 of 28
Mx	4 (11)	NRAS	5 of 11
Primary site		KRAS	1 of 7
Nasal cavity	32 (84)	Pigmentation	
Paranasal sinuses	6 (16)	Melanotic	24 (63)
		Amelanotic	14 (37)

Not all tumors were subjected to mutational analysis. Mx: Complete staging including thoracoabominal imaging was dispensed in three cases, due to a palliative situation. Furthermore, there was one case with an unclear hepatic lesion denying further diagnosis and therapy

Histopathological analysis revealed slightly more melanotic (63%) compared to amelanotic tumors (37%). 28 tumors were subjected to sequencing (*n* = 22; 58% Sanger Sequencing and *n* = 6; 16% Next Generation Sequencing). None of the 28 analyzed tumors showed a KIT mutation. Five patients had a mutation of the NRAS-gene, four in exon 3 (exon 3 position 181 cytosine to adenine (c. 181C > A; p.Q61K) and one in exon 2 (exon 2 position 35 guanine to adenine (c.35G > A; p.G12D). One out of 22 tumors analyzed had a class 3 (kinase-impairing) BRAF mutation (BRAF (Exon15): c.1780G > A; p.D594N), while no “classic” activating BRAFV600 mutation was found. An activating mutation of the KRAS-gene (KRAS: Exon 3: c.138A > C; p.Q61H) was detected in one of seven tumors analyzed (Table 1). No mutations could be detected in other genes analyzed.

Thirty-one patients were treated in curative intention: either by surgery, primary radiotherapy or ICI (Table 2). Twenty-one patients were treated by surgery with the goal of gross-total resection (GTR). In seven cases only cytoreductive surgery (CRS) could be achieved, followed by a primary radiotherapy (pRT). Selective neck dissection was performed in four patients due to clinically and radiologically suspect lymph nodes. Three patients received a primary combination therapy of nivolumab and ipilimumab upon their request. One patient suffering from a T3 tumor showed a histologically proven complete response (CR) after 4 cycles of nivolumab (1 mg per kilogram of body weight (mg/kg bw)) and ipilimumab (3 mg/kg bw) treatment and received no further surgical treatment. Until now, no local recurrence was found for 29 months. Two patients showed progressive disease (PD) following four and two cycles of nivolumab (3 mg/kg bw) and ipilimumab (1 mg/kg bw) combination therapy analogue to the OpacinNeo trial [26], respectively. Ten surgically treated patients received an adjuvant therapy: nine by adjuvant radiotherapy and one by adjuvant radiotherapy plus ICI with nivolumab (480 mg q4w).

**Table 2:** Treatment regimens of 31 patients treated in a curative intent

*T*	*n*	Primary ICI	Surgery	CRS + pRT	Margin status		Adjuvant treatment		
					GTR	R2	RT	RT + ICI	None
T3	18	2	14	2	14	2	5	0	11
T4a	8	1	4	3	4	3	1	1	5
T4b	5	0	3	2	3	2	3	0	2
Total	31	3	21	7	21	7	9	1	18

*CRS* cytoreductive surgery; *GTR* gross total resection; *ICI* immune checkpoint inhibition, *pRT* primary radiotherapy; *RT* radiotherapy

There were seven palliative cases at the time point of primary diagnosis (Table 3). Three patients with an extensive tumor burden and dementia received primary palliative treatment in terms of best supportive care. The other four patients had distant metastases and underwent CRS of the primary tumor along with a selective neck dissection in two of these cases due to clinically and radiologically suspect lymph nodes. One of these patients received a primary radiotherapy of the sinonasal tumor region and the regional lymphatic pathways with an overall dose of 59.4 Gy for further local control. Two patients refused or were not eligible for further treatment and two received an additional systemic palliative treatment. One patient had to be referred to best supportive care following four cycles of ipilimumab (3 mg/kg bw q3w), 25 cycles of nivolumab (3 mg/kg bw q2w), one cycle of dacarbacine (250 mg/m^2^ KOF), 25 cycles of dacarbacine (125 mg/m^2^ KOF) and two cycles of a combination therapy of relatlimab and nivolumab according to the CA224-020 protocol. The other patient died shortly after initiation of palliative chemotherapy with dacarbacine (250 mg/m^2^ KOF).

**Table 3:** Treatment regimens of seven patients in a primary palliative setting

*T*	*n*	BSC	Surgery	CRS + pRT	Medical treatment	
					ICI	CT
T3	1	0	1	0	0	0
T4a	1	0	1	0	0	1
T4b	5	3	1 (CRS)	1	1	1
Total	7	3	3	1	1	2

Two patients received further palliative systemic treatment due to local residual disease and distant metastases

*GTR* gross total resection; *primary RT* primary radiotherapy; *CRS* cytoreductive surgery; *ICI* immune checkpoint inhibition; *CT* chemotherapy

Follow-up data were collected from clinical records. A complete follow-up was possible in 35 patients (92%). Information about OS was available for all patients. Follow-up staging data of two patients were incomplete for regional and distant metastases and in one patient for distant metastases only. These patients left follow-up due to an advanced age or a palliative situation. Median follow-up time was 33 months (range 3–115 months). There were 31 patients with completion of first line treatment in curative intent. One patient died from meningoencephalitis caused by Varicella zoster virus shortly after completing adjuvant radiotherapy. As there is no information about residual or recurrent disease, this case was not included into the analysis of the follow-up data, leaving 30 patients for analysis. After initial treatment, residual disease was present within the primary site in five (17%) cases and recurrent disease in 13 patients (43%). Only three (10%) patients remained free of recurrence during follow up. Out of 30 patients treated in curative intent regional recurrence was observed in six cases (20%) and distant recurrence in 20 (67%) (Table 4). In the whole study population of 38 patients, nine developed regional lymph node metastases and 25 presented distant metastases during the whole course of the disease. Lung (15 cases) and liver (8 cases) were the most commonly affected organs for metastases.

**Table 4:** Follow-up data regarding recurrent or residual disease of 30 patients treated in a curative intent

*T*	*n*	Local residual disease	No recurrence	Local recurrence	Regional recurrence	Distant recurrence	Incomplete data
T3	18	3	3	10	3	10	2
T4a	7	0	0	2	2	6	0
T4b	5	2	0	1	1	4	1
Total	30	5	3	13	6	20	3

Post-treatment staging data are incomplete for three patients. Patients suffering from recurrence at different sites are mentioned accordingly in each of the last three columns

Most common therapeutic approaches for recurrent or residual disease including distant metastases were chemotherapy and ICI, each in 11 patients followed by radiotherapy and surgery, each in six cases. Out of 11 ICI patients, eight received ICI monotherapy only (four cases of pembrolizumab; two cases of nivolumab; two cases of ipilimumab), two patients received ICI monotherapy (one case of pembrolizumab; one case of ipilimumab followed by nivolumab) followed by a combination therapy of nivolumab and ipilimumab in one case and relatlimab (Anti-LAG-3 antibody) and nivolumab in the other case. One patient received a combination therapy of ipilimumab and nivolumab only. Of these 11 cases, seven progressed while stable disease could be observed in one case and CR in three patients. In the patient with stable disease, pembrolizumab (2 mg/kg bw every 3 weeks) was initiated due to unspecific pulmonary nodules diagnosed after cT3 primary tumor excision and adjuvant radiotherapy. Therapy had to be discontinued after two cycles due to autoimmune hepatitis. After 37 months, the patient progressed in terms of a local recurrence, which could be removed surgically. No regional or distant metastases could be observed for 41 months so far. There was one patient with no evidence of disease for more than 5 years following surgical excision of mediastinal lymph node metastases and 42 cycles of nivolumab therapy (3 mg/kg every 2 weeks) for another retroperitoneal node. Another patient developed local recurrence and pulmonary metastases 18 months after primary therapy. The sinonasal tumor was excised and pembrolizumab (2 mg/kg bw every 3 weeks) was initiated. Following 29 cycles of therapy an ongoing CR could be observed for more than 5 years. In the third case, the patient developed a cervical node metastasis, bone metastases of the skull as well as soft tissue metastases of the abdominal wall and mamma 11 months after primary therapy. The metastasis of the mamma was excised for histological analysis and a partial remission of the residual masses could be achieved following three cycles of nivolumab (1 mg/kg bw) and ipilimumab (3 mg/kg bw). However, ICI was discontinued due to severe neuropathy. After 9 months a new axillary lymph node metastasis was excised and after four additional months a retroperitoneal metastasis was treated by radiotherapy. Interestingly, a complete remission of all residual disease was observed thereafter and there is no evidence of cancer for 19 months so far.

**Fig. 1:**
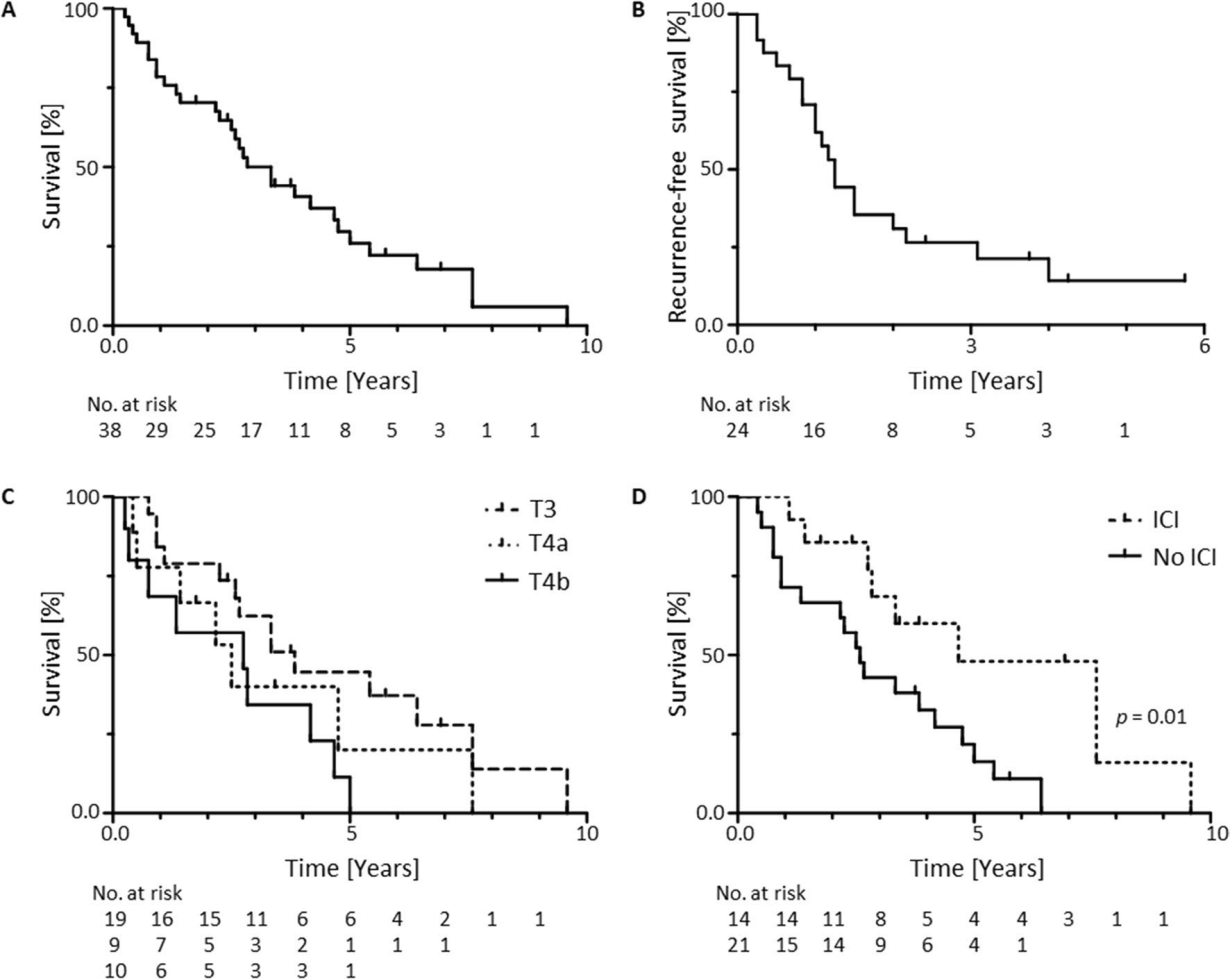
Survival data. **A** Overall survival (OS) and **B** recurrence free survival of all 38 patients analysed. **C** OS of 19 cases with a T3 tumor, 9 cases with a T4a tumor and 10 cases with a T4b tumor. **D** Comparison of OS of 14 patients receiving (ICI) and 21 patients not receiving (no ICI) ICI treatment during the course of disease

Median OS for all patients was 40 months, with a 2-year OS-rate of 70% and a 5-year OS-rate of 26% (Fig. 1A). Median RFS was 15 months, with a 2-year RFS-rate of 30% and a 5-year RFS-rate of 14% (Fig. 1B). OS for T3 tumors was significantly better compared to T4b tumors (*p* = 0.04; Fig. 1C). Interestingly, OS was significantly better for patients who received ICI during course of the disease compared to patients who did not receive ICI (*p* = 0.01; Fig. 1D).

## Discussion

Mucosal melanoma is an aggressive disease with a high mortality rate. Mucosal and CMM behave dissimilarly in terms of biology, clinical presentation and outcomes. Compared to CMM, survival rates are significantly lower. 5-year OS is 26-45% for mucosal melanoma but 39-97% for CMM [9]. An additional difference to CMM is that patients suffering from mucosal melanoma often present with locally advanced disease. Ozturk et al. reported a localized manifestation in 55% of patients with mucosal melanomas whereas 22% had regional and 23% distant metastases [26]. In our cohort, 50% had a localized primary tumor and only 13% presented with regional and 11% with distant metastases. However, 25 patients developed distant metastases during the course of disease. In line with the literature, the most common sites of metastases in our cohort are liver and lungs [9]. Consistent with clinical features, genetic examination of mucosal and CMM also reveals marked differences. Driver mutations such as in BRAF gene occur in up to 66% of CMM [27], but only in up to 8.4% of SNMM [28]. In our cohort, we observed a non-BRAF V600 mutation in only one case (4.5%). Other reported mutations such as in KIT genes can be detected at an estimated frequency of 6.4% and 8.6% [26, 29]. Further mutations can be detected in NRAS genes and NF1. As prognosis of SNMM remains poor, genetic analyses in the BRAF and c-KIT genes should be intensified to detect such few cases with a targetable genetic mutation. In line with the literature, we could identify very few mutations in NRAS. A mutation in the c-KIT gene was not detected in this cohort.

Due to the low incidence and hence limited evidence, there is no guideline for standard therapy of SNMM. Until now, surgery with negative margins followed by an adjuvant radiotherapy is the most accepted curative measure for SNMM. The surgical approaches include endoscopic surgery, open resection and combinations of both. The therapeutic algorithm of our cohort differs between patients due to the long period analyzed. For the past few years, the standard procedure at our department is the interdisciplinary evaluation of each case. In case of surgery, tumors are operated using an open approach combined with a microscopic and/or endoscopic technique followed by an adjuvant radiotherapy. Interestingly, there is increasing evidence that endoscopic compared to open approaches do not adversely affect outcome or may even be beneficial regarding local control and survival rates [11, 30]. In our cohort, twenty-eight patients were treated surgically and in 22 cases a gross total resection could be achieved. A complete surgical resection can be challenging due to the anatomical location and radical surgery may increase morbidity or reduce patients’ quality of life. However, Heppt et al. showed an increased risk for a local recurrence in case of margin-positive resection and locoregionally advanced tumors [29]. Ganti and colleagues have evaluated the National Cancer Database for treatment modalities of 1847 patients with SNMM. They could show that surgery with clear margins prolonged the survival rate [31]. A benefit of clear margins on RFS and OS was also shown in further retrospective single center studies [10, 11, 14] and a recent retrospective analysis of the National Cancer Database [32]. In contrast, there are other studies showing that surgical radicality does not correlate with patient survival in anorectal and female genital melanoma as well as SNMM [12, 13, 33–35]. Regardless of this, surgery may help to improve local control [33, 36] and subsequently to prevent local complications such as tumor hemorrhage or tumor extension into adjacent structures such as the orbit or the skull base.

Ganti and colleagues could also identify advanced age, T4 status and distant metastases as factors associated with an impaired prognosis [31]. In line, Liu et al. showed that distant metastases were a dominant predictor for a poor prognosis in a retrospective analysis of 51 patients [37]. Furthermore, Moya-Plana et al. stated, that even early-stage mucosal melanoma of the head and neck are associated with poor oncologic outcome due to distant metastases independent of the margin resection status [38]. Low et al. showed that the presence of regional and distant metastases at time of diagnosis is a negative prognostic factor regarding OS and disease-specific survival in SNMM [39]. Therefore, reducing the risk of distant metastases is likely to be helpful to improve survival. As response rates of SNMM to chemotherapy are poor, ICI might be a potent therapeutic option. In a multicenter retrospective analysis of 505 SNMM patients ICI was shown to improve survival of recurrent or persistent disease, with or without distant metastasis [40]. Ganti and colleague’s retrospective database analysis could show, that patients with metastatic SNMM showed a survival benefit from ICI. In cuMM a paradigm shift from chemotherapy to the use of monoclonal antibodies against PD-1 or CTLA-4 has taken place during recent years [18, 41]. Interestingly, there is also data indicating efficacy of ICI in mucosal melanoma although response rates seem to differ between mucosal and CMM. In 2017, D’Angelo et al. conducted a pooled analysis from prospective clinical trials of unresectable stage III or stage IV mucosal melanomas in terms of survival rate after first line treatment with nivolumab alone versus combination therapy of nivolumab and ipilimumab [21]. The objective response rates of 23.3% was significantly lower compared to CMM. The combination therapy appeared to be slightly more effective than monotherapy. However, this enhancement is accompanied by an increase in toxicity. Shoushtari et al. examined 35 patients with mucosal melanoma treated with anti-PD-1 monotherapy. They were able to show an ORR of 23% in 35 patients. The median PFS was 3.9 months [24]. The potential causes of worse response rate of ICI in mucosal melanomas are intensively discussed. One cause may be the low expression of PD-L1 in mucosal melanoma. D’Angelo and colleagues could show by immunohistochemistry that only 21% of the 121 mucosal melanomas were positive for PD-L1. Second, a low mutational burden of mucosal melanomas could explain the reduced response rate to ICI. CMM exhibit a mutational signature associated with UV damage, which is favorable for ICI therapy. This is not the case in mucosal manifestations.

With the approval of ICI for SNMM, ICI became an integral part for the treatment of patients in a palliative setting at our institution. Thus, all palliative patients from then on received ICI as part of their therapy. Furthermore, there were three patients receiving ICI as a primary and one as an adjuvant therapy. Overall, 15 patients received ICI in different settings regarding time point, duration, mono or double ICI. When comparing these patients to those who did not receive ICI, ICI was associated with an improved overall survival. As a matter of course, this analysis possesses challenges such as the comparison of two heterogenous groups, the low number of patients and a long period of 21 years evaluated. Eleven patients received ICI as part of a palliative therapy. Seven cases progressed under ICI while CR could be observed in three cases following nivolumab or pembrolizumab therapy and PR in one case following nivolumab and ipilimumab combination therapy.

Possible future approaches could be neoadjuvant concepts or the early initiation of an adjuvant systemic therapy to prevent distant metastases as an initial therapeutic step. Data on neoadjuvant therapy with ICI in SNMM are missing. In our cohort, three patients received ICI in a primary setting. In one case, a CR was seen without local tumor recurrence for 29 months so far but in two cases, significant tumor progression was observed. Tumor progression in the paranasal sinus system can rapidly result in life-threatening situations by infiltration of vital organs such as the brain or the carotid artery. To the opinion of the authors, surgical therapy with or without radiotherapy is useful for a rapid local tumor control. Nevertheless, an early therapy with ICI could be an option to minimize the risk of local recurrence and distant metastases. Therefore, primary surgical therapy followed by an adjuvant radiotherapy along with a rapid initiation of an adjuvant ICI could benefit patients. However, it remains unclear how to design adjuvant therapy regimes regarding duration, mono or double ICI. Further evidence is needed to clarify the impact of an early adjuvant ICI in SNMM for local and distant control.

According to the literature, the risk of local recurrence in SNMM is up to 50% [42]. Adjuvant radiotherapy was shown to improve local control [13, 17, 36, 40, 43]. However, benefit of adjuvant radiotherapy in SNMM is still under discussion. Until now, there is no clear evidence for a positive effect of adjuvant radiotherapy on OS [30, 33, 36].

## Conclusions

There is increasing evidence for the efficacy of ICI in SNMM patients even though response rates seem to be lower than in CMM. Surgery with adjuvant radiotherapy helps to provide local control. However, local recurrence is a possible threat. Furthermore, distant failure is predominant in SNMM and is associated with a dismal survival. Early adjuvant ICI may improve distant control rates of occult systemic disease as a major priority and could help to prevent local recurrences. Therefore, future studies should critically examine concepts of an early adjuvant ICI following surgery and adjuvant therapy.

## Data Availability

The data presented in this study are available on request from the corresponding author. The data are not publicly available due to reasons of legal data protection and ethical restrictions.

## Abbreviations

SNMM: Sinonasal mucosal melanoma
CMM: Cutaneous malignant melanoma
OS: Overall survival
ICI: Immune checkpoint inhibition
PD-1: Programmed Cell Death Protein 1
CTLA-4: Cytotoxic T-lymphocyte-associated Protein 4
ORR: Objective response rates
PFS: Progression-free survival
GTR: Gross total resection
CRS: Cytoreductive surgery
pRT: Primary radiotherapy
PD: Progressive disease
CR: Complete response
RFS: Recurrence-free survival
mg/kg bw: Milligram per kilogram of body weight
